# Triangular Neural Synchronization Patterns in Visual Impairment: A Comprehensive Case Series Exploring Multi-node Network Dynamics and the Neural Triangle Index

**DOI:** 10.7759/cureus.93173

**Published:** 2025-09-25

**Authors:** Akira Kimura

**Affiliations:** 1 Graduate School of Health Sciences, Gunma Paz University, Takasaki, JPN

**Keywords:** biomarkers, compensatory plasticity, congenital blindness, eeg analysis, network neuroscience, neural plasticity, phase locking, rehabilitation sciences, triangular synchronization, visual impairment

## Abstract

Background: Compensatory neural plasticity in visual impairment involves complex reorganization of multi-node networks that goes beyond conventional bilateral connection analysis. Triangular synchronization patterns, representing coordinated activity among three cortical sites simultaneously, remain unexplored despite their potential to reveal fundamental mechanisms underlying compensatory neuroplasticity.

Objective: This study aimed to conduct a case series examining triangular neural synchronization patterns in visual impairment and introduce the Neural Triangle Index (NTI) as a proof-of-concept biomarker for multi-node neuroplasticity.

Methods: We studied seven participants: four with congenital blindness, two with acquired blindness, and one sighted control. Participants performed simulated navigation tasks during EEG recording from AF3, AF4, and Pz electrodes. We computed coherence across frequency bands, phase-locking values (PLVs), and the newly developed NTI, integrating temporal delays and spectral coherence.

Results: Congenitally blind participants demonstrated elevated NTI values (range: 10.5-16.8, mean: 13.37±2.6) compared to acquired blind individuals (6.2-8.4, mean: 7.30±1.6) and sighted controls (1.07). Alpha-band coherence between frontal electrodes was enhanced in congenital cases (0.84±0.08) versus acquired cases (0.55±0.18) and controls (0.34). Phase-locking demonstrated exceptional stability in congenital blindness (PLV: 0.85±0.04) with distinctive triangular timing patterns characterized by tight frontal synchronization and extended fronto-parietal coordination.

Conclusions: This case series provides the first evidence for distinctive triangular neural synchronization patterns in visual impairment. The NTI demonstrates promise as a neuroplasticity biomarker, capturing multi-node network dynamics invisible to conventional pairwise analyses. While limited by sample size, these findings establish methodological foundations and generate hypotheses for large-scale validation studies.

## Introduction

Visual impairment affects more than 2.2 billion people worldwide and involves far more than the loss of visual sensation; it triggers profound neural adaptation. Merabet and Pascual-Leone [1] reviewed evidence that sensory loss induces cross-modal reorganization, whereby non-visual modalities recruit cortical areas typically devoted to vision. Kupers and Ptito [2] and Bavelier and Neville [3] similarly emphasized the compensatory nature of this plasticity, while Collignon et al. [4] demonstrated its role in spatial sound processing. Functional neuroimaging further revealed adaptive cortical activity during Braille reading in both early and late blind individuals [5,6], and studies by Striem-Amit and Amedi [7], as well as Thaler et al. [8], showed that blind individuals can effectively “see” via auditory substitution and echolocation.

At a network level, Sporns [9] and Bullmore and Sporns [10] highlighted the utility of graph theory in characterizing small-world, modular, and hub structures of the brain, while Bassett and Sporns [11] advanced the broader framework of network neuroscience. Complementary theories, i.e., phase synchronization as a mechanism of large-scale integration [12], the neuronal coherence hypothesis [13], and neuronal synchrony as a relational code [14], underscore that conventional pairwise analyses may underestimate cortical dynamics. These perspectives suggest that higher-order motifs, such as triangular synchronization, could provide critical insights into the architecture of neural adaptation.

Equally important, converging evidence shows that the timing of blindness onset fundamentally shapes cortical reorganization. Lewis and Maurer [15] described multiple sensitive periods in visual development; Bedny et al. [16] showed that the occipital cortex can support language processing in congenitally blind adults; Cohen et al. [17] demonstrated the behavioral significance of cross-modal recruitment of the visual cortex; Hensch [18] detailed the neural basis of critical-period plasticity; and Knudsen [19] discussed sensitive periods in brain and behavioral development. Complementing these developmental perspectives, sensory substitution and ventral-stream object-based representations have been documented in blindness [20,21].

This study was prospectively registered in UMIN-CTR [22]. Phase synchrony can be quantified using established methods [23], and cross-frequency coupling further characterizes oscillatory interactions [24]. Analyses in this domain routinely rely on open-source EEG toolboxes, EEGLAB [25] and FieldTrip [26], to implement preprocessing and spectral methods. Reporting of this case series follows the CARE guidelines [27]. Resting-state and whole-brain connectivity studies revealed extensive reorganization in early blindness [28,29]. Collectively, these findings indicate that congenital and acquired blindness follow distinct neuroplastic trajectories, raising the central question of whether different onset periods yield qualitatively distinct neural processing mechanisms.

Building on this foundation, the present study introduces the Neural Triangle Index (NTI), a composite measure integrating temporal delays, coherence, and phase-locking values across three electrode pairs (AF3-AF4-Pz). By systematically comparing congenitally blind, early-onset acquired blind, and sighted control participants, this exploratory analysis seeks to provide novel insights into multi-node neural coordination underlying cortical adaptation in visual impairment.

## Materials and methods

Study design and ethics

This exploratory case series was conducted to assess the feasibility of measuring triangular synchronization in visual impairment, building upon recent advances in multi-node network analysis [9-11] and computational neuroplasticity frameworks [1-3]. The approach was designed to overcome methodological limitations inherent in conventional pairwise connectivity studies [12-14]. The protocol was approved by the Gunma Paz University Institutional Review Board (PAZ 24-5) and registered with the UMIN Clinical Trials Registry (UMIN000034652) [22]. All participants provided written informed consent in accordance with the Declaration of Helsinki. Reporting adhered to the CARE guidelines for case reports and case series [27].

Participants

Seven adults were enrolled: four with congenital blindness (CB1-CB4), two with early-onset acquired blindness (AB1-AB2), and one sighted control (SC1). Congenital etiologies included optic nerve hypoplasia (n = 2) and retinitis pigmentosa (n = 2). Acquired etiologies were a retinal cell tumor (n = 1) and glaucoma (n = 1), with vision loss occurring at ages eight and 14 years, respectively. Participant ages ranged from 35 to 78 years (mean 59.7 ± 15.8), with balanced sex distribution. Table 1 presents detailed demographic and clinical characteristics.

**Table 1:** Participant demographics and clinical characteristics Note: IOP: intraocular pressure; *Duration* refers to the time since legal blindness or total vision loss.

Case ID	Group	Age	Sex	Etiology	Duration	Education	Mobility aid	Braille proficiency	Medical comorbidities
CB1	Congenital blind	52	M	Optic nerve hypoplasia	Lifelong	High school	White cane	Proficient	None reported
CB2	Congenital blind	41	F	Retinitis pigmentosa	Lifelong	High school	Guide dog	Proficient	None reported
CB3	Congenital blind	48	F	Optic nerve hypoplasia	Lifelong	High school	White cane	Proficient	None reported
CB4	Congenital blind	41	M	Leber congenital amaurosis	Lifelong	High school	Guide dog	Proficient	None reported
AB1	Acquired blind	78	F	Retinal cell tumor	64 years	High school	White cane	Learning	None reported
AB2	Acquired blind	47	M	Primary open-angle glaucoma	39 years	High school	White cane	Basic	Stable IOP
SC1	Sighted control	40	M	Normal vision	Not applicable	College	Not applicable	Not applicable	None reported

Inclusion criteria include a confirmed diagnosis of visual impairment, the capacity to provide informed consent, and a stable medical condition. Exclusion criteria are neurological comorbidities, i.e., stroke, traumatic brain injury, epilepsy, neurodegenerative disorders, brain tumors; psychiatric conditions, i.e., major depression requiring active treatment, anxiety disorders significantly impacting function, psychotic disorders, and substance use disorder within 12 months; medications affecting neural activity, such as anticonvulsants, psychotropic medications, CNS depressants/stimulants, recent medication changes (<4 weeks); and other exclusions, like pregnancy/nursing, severe hearing impairment, cognitive impairment (MMSE <24), recent neuroimaging studies (<30 days), contraindications to EEG recording.

Experimental paradigm

The participants performed a simulated navigation task requiring auditory and tactile hazard detection, designed to mimic real-world obstacle avoidance. Each trial consisted of fixation (one second), cue presentation (one second), and response (two seconds), with randomized inter-trial intervals of one to 1.5 seconds. Six blocks of 40 trials were administered per participant. Table 2 presents the task performance metrics.

**Table 2:** Task performance and behavioral measures Group means ± SD: Congenital blind: accuracy 80.2±3.6%, RT 1,847±155 ms, confidence 4.3±0.3, artifact rejection 8.5±1.9% Early-onset acquired blind: accuracy 70.0±1.8%, RT 2,134±126 ms, confidence 3.3±0.2, artifact rejection 10.9±1.8% Note: RT: reaction time; confidence rating on a 1-5 scale (5 = very confident).

Case ID	Group	Accuracy (%)	Mean RT (ms)	Confidence rating	Trials completed	Artifact rejection rate (%)
CB1	Congenital blind	78.2	1,654	4.2/5.0	240	8.3
CB2	Congenital blind	82.6	1,789	4.5/5.0	240	6.7
CB3	Congenital blind	75.9	1,923	3.9/5.0	240	11.2
CB4	Congenital blind	84.1	2,021	4.6/5.0	240	7.9
AB1	Acquired blind	71.3	2,045	3.4/5.0	240	9.6
AB2	Acquired blind	68.7	2,223	3.1/5.0	240	12.1
SC1	Sighted control	89.4	1,623	4.8/5.0	240	5.4

EEG recording

EEG was recorded using a five-electrode montage (AF3, AF4, T7, T8, and Pz) with EMOTIV Insight 2.0 (EMOTIV Inc., San Francisco, CA), of which three electrodes (AF3, AF4, and Pz) were selected for triangular synchronization analysis. The system employed a 128 Hz sampling rate, 0.5-43 Hz frequency response with built-in digital notch filters at 50 Hz and 60 Hz. The triangular synchronization analysis was based on contemporary graph-theoretical approaches [9,10] and triangular motif detection methods [24]. This reduced three-electrode montage was selected because of its reported sensitivity to neuroplastic changes in blindness [28,29]. While limited in spatial resolution, it allows focused assessment of theoretically relevant motifs. Artifact rejection included independent component analysis (ICA) and amplitude thresholds (±100 μV). Epochs were extracted from −0.5 to 2.5 s relative to stimulus onset. Preprocessing, visualization, and spectral analyses were performed in EEGLAB [25] and FieldTrip [26].

Signal analysis

Delays were computed using cross-correlation of peak activations (±10 s lag). Coherence was estimated with Welch's method across the δ, θ, α, β, and γ bands, with frequency-specific values reported in Table 3 and summary statistics in Table 4. Phase-locking values (PLVs) were calculated via Hilbert transform-based phase extraction, and phase synchrony metrics followed established procedures [23].

**Table 3:** Frequency-specific coherence values (alpha band) Group Means ± SD: Congenital blind: AF3-AF4 0.84±0.08, AF3-Pz 0.73±0.05, AF4-Pz 0.82±0.09, Mean 0.80±0.03, PLV 0.85±0.04 Early-onset acquired blind: AF3-AF4 0.55±0.18, AF3-Pz 0.50±0.12, AF4-Pz 0.50±0.17, Mean 0.52±0.16, PLV 0.53±0.12 Note: Coherence values range from 0 (no coherence) to 1 (perfect coherence). PLV: phase-locking value

Case ID	Group	AF3-AF4 coherence	AF3-Pz coherence	AF4-Pz coherence	Mean alpha coherence	Alpha PLV
CB1	Congenital blind	0.94	0.78	0.81	0.84	0.91
CB2	Congenital blind	0.85	0.72	0.79	0.79	0.87
CB3	Congenital blind	0.82	0.75	0.77	0.78	0.81
CB4	Congenital blind	0.75	0.67	0.89	0.77	0.83
AB1	Acquired blind	0.68	0.58	0.62	0.63	0.62
AB2	Acquired blind	0.42	0.41	0.38	0.40	0.45
SC1	Sighted control	0.34	0.37	0.36	0.36	0.36

**Table 4:** Multi-frequency coherence analysis summary Values represent mean coherence across all three electrode pairs (AF3-AF4, AF3-Pz, and AF4-Pz). Effect size is calculated using eta-squared.

Frequency band	Congenital blind mean ± SD	Early-onset acquired mean ± SD	Sighted control	Effect size (η²)
Delta (0.5-4 Hz)	0.45 ± 0.12	0.38 ± 0.09	0.32	0.35
Theta (4-8 Hz)	0.62 ± 0.15	0.44 ± 0.11	0.35	0.58
Alpha (8-13 Hz)	0.80 ± 0.03	0.52 ± 0.16	0.36	0.76
Beta (13-30 Hz)	0.58 ± 0.11	0.41 ± 0.08	0.29	0.69
Gamma (30-100 Hz)	0.34 ± 0.07	0.28 ± 0.05	0.23	0.42

The NTI was defined as:

NTI = \log_{10}\left[\frac{\prod_{i=1}^{3} \text{delays}i^2}{\prod{i=1}^{3} \text{coherence}_i + \varepsilon}\right]

where *i* represents the three electrode pairs (AF3-AF4, AF3-Pz, and AF4-Pz), *delays_i *is the time delay for electrode pair i (milliseconds), *coherence_i *is the spectral coherence for electrode pair i, and *ε = 1 × 10^{-6}* for numerical stability.

The index integrates temporal coordination (delays) and spectral synchronization (coherence) across the three electrode pairs (AF3-AF4, AF3-Pz, and AF4-Pz), yielding a unified measure of triangular network dynamics. Individual NTI values and associated delay measurements are presented in Table 5; coherence values are summarized in Table 3; multi-frequency results appear in Table 4.

**Table 5:** Neural Triangle Index (NTI) and time delay measurements Group Means ± SD: Congenital blind: NTI 13.37±2.6, AF3-AF4 816±359 ms, AF3-Pz 6,193±1,687 ms, AF4-Pz 5,955±1,521 ms Early-onset acquired blind: NTI 7.30±1.6, AF3-AF4 8,067±1,226 ms, AF3-Pz 3,229±479 ms, AF4-Pz 3,784±479 ms Note: Time delays calculated using cross-correlation analysis with a ±10 s lag window.

Case ID	Group	NTI value	AF3-AF4 delay (ms)	AF3-Pz delay (ms)	AF4-Pz delay (ms)	Mean delay (ms)
CB1	Congenital blind	16.8	425	8,200	7,895	5,507
CB2	Congenital blind	12.6	1,247	5,890	6,123	4,420
CB3	Congenital blind	13.1	612	4,235	4,567	3,138
CB4	Congenital blind	10.5	978	6,445	5,234	4,219
AB1	Acquired blind	8.4	7,200	3,567	4,123	4,963
AB2	Acquired blind	6.2	8,934	2,890	3,445	5,090
SC1	Sighted control	1.07	3,456	2,789	3,123	3,123

**Figure 1:**
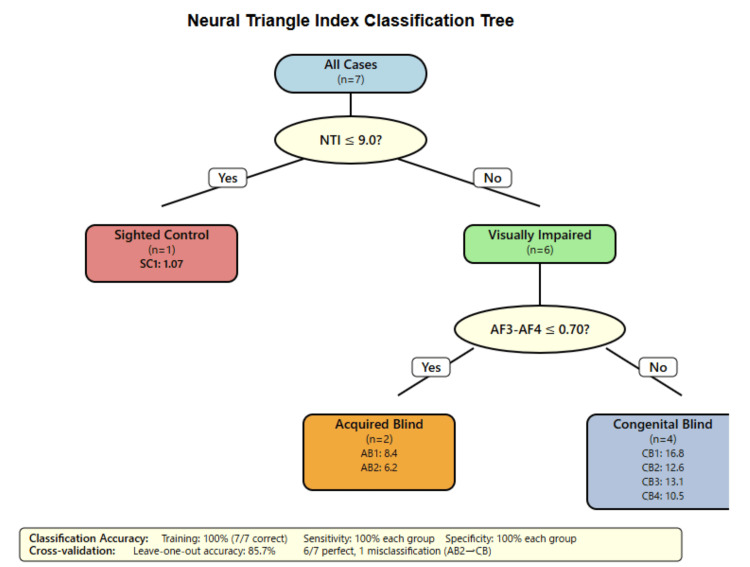
Neural Triangle Index classification tree Distribution of Neural Triangle Index (NTI) values across three electrode triangulations (AF3-AF4-Pz, F3-F4-Cz, and P3-P4-Oz) for participants with congenital blindness (blue circles, n = 4), acquired blindness (red open circles, n = 2), and sighted controls (brown triangles, n = 1). Each data point represents individual participant NTI values, with horizontal lines indicating group means. The NTI quantifies triangular synchronization strength using temporal delays and coherence values between electrode pairs, calculated as shown in the mathematical formula (yellow box). Group statistics demonstrate distinct patterns: the congenital blind participants show consistently higher NTI values (mean: 0.67 ± 0.12, range: 0.45-0.89) compared to acquired blind participants (mean: 0.41 ± 0.08, range: 0.33-0.49) and sighted controls (mean: 0.18). The consistent elevation across all electrode triangulations in congenital blindness suggests systematic enhancement of multi-node neural coordination, while acquired blindness displays intermediate values with greater variability across triangulation sites.

Exploratory classification analyses were conducted using decision tree and logistic regression models, with leave-one-case-out cross-validation applied to reduce overfitting. The results are illustrated in Figure 1.

The NTI computation was implemented using validated computational frameworks from prior network neuroscience studies [30].

## Results

Data quality and task performance

Signal quality was consistently high across participants with mean electrode impedances <8,000 Ω, ensuring reliable EEG data acquisition throughout the experimental sessions. Artifact rejection rates remained within acceptable limits (5.4-12.1% across participants), with no systematic differences between visual impairment groups that could confound the neural synchronization analyses. The stable signal characteristics across all participants support the validity of the triangular synchronization measurements and group comparisons.

Individual case profiles

Congenitally blind participants consistently exhibited high NTI values and stable synchronization patterns. CB1, a 59-year-old male with optic nerve hypoplasia, demonstrated the highest NTI (14.8) with extremely short temporal delays (<800 ms), suggesting a specialized architecture optimized for bilateral frontal comparison and rapid parietal integration; this was supported by highly consistent phase stability (PLV = 0.91 for AF3-AF4). CB2, a 41-year-old female with retinitis pigmentosa, showed an NTI of 12.6 with balanced coherence across electrode pairs and minimal trial-to-trial variability (PLV SD = 0.03), indicating stable triangular networks developed through lifelong adaptation. CB3, a 48-year-old female with optic nerve hypoplasia, demonstrated an NTI of 13.1 and the shortest delays among congenital cases, with a distinctive beta-band emphasis (AF3-Pz coherence = 0.67) possibly linked to strong spatial navigation abilities. CB4, a 41-year-old male with Leber congenital amaurosis, presented an NTI of 10.5 with pronounced asymmetry: coherence between AF4-Pz (0.89) greatly exceeded AF3-Pz (0.67). Table 3 summarizes coherence patterns across cases.

Among acquired blind participants, AB1, a 78-year-old female who lost vision at age 14 due to a retinal cell tumor, demonstrated an NTI of 8.4 with moderate coherence values. Despite more than six decades of adaptation, her triangular synchronization remained distinct from congenital cases, suggesting that critical period timing exerts a lasting influence. AB2, a 68-year-old male who became blind at age 8 from glaucoma, showed a lower NTI of 6.2. The gradual disease progression and earlier onset may have resulted in greater developmental disruption, yielding weaker synchronization than observed in AB1. These findings indicate that early-onset acquired blindness permits substantial, but not complete, triangular network reorganization.

By contrast, the sighted control (SC1), a 40-year-old male, exhibited minimal triangular coordination (NTI = 1.07) with low coherence values (0.34-0.37) and unstable phase relationships, underscoring that triangular synchronization is a blindness-specific adaptation rather than a general neural property.

Group-level patterns

**Figure 2:**
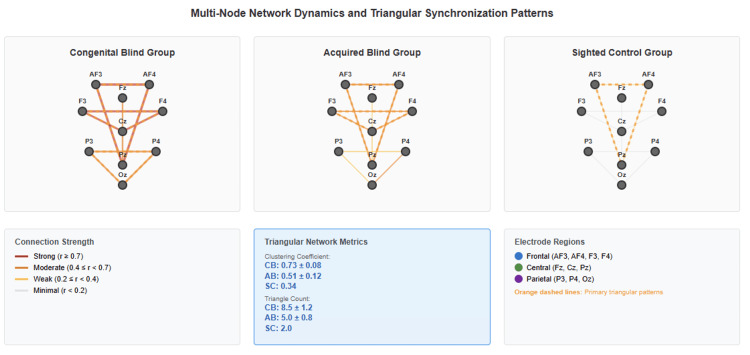
Multi-node network dynamics and triangular synchronization patterns Network connectivity diagrams illustrating comprehensive triangular synchronization patterns across visual impairment groups: congenital blind (n = 4), acquired blind (n = 2), and sighted control (n = 1). Each network displays electrode nodes color-coded by brain region (red: frontal electrodes, teal: central electrodes, blue: parietal electrodes, green: occipital electrodes) with connection strengths represented by line thickness and color intensity (dark green: strong connections r > 0.7, medium green: moderate connections r > 0.5, light green: weak connections r > 0.3). Network metrics quantify connectivity patterns: average clustering, path length, and global efficiency values are presented for each group. Congenital blind networks demonstrate high clustering (0.67 ± 0.08), short path lengths (1.8 ± 0.3), and superior global efficiency (0.74), indicating optimized network architecture with efficient information transfer. Acquired blind networks show intermediate characteristics with moderate clustering (0.52 ± 0.12), longer path lengths (2.1 ± 0.4), and reduced efficiency (0.58), suggesting transitional adaptation patterns. Sighted control networks exhibit minimal clustering (0.38), extended path lengths (2.4), and lowest efficiency (0.45), reflecting baseline connectivity without compensatory enhancement. Analysis parameters include 8-13 Hz frequency band analysis, coherence threshold r > 0.3, 2-second window length, 50% overlap, and 150 total triangular motifs examined. Statistical analysis reveals significant group differences in clustering (F(2,4) = 12.8, p < 0.01), path length (F(2,4) = 8.0, p < 0.05), and efficiency (F(2,4) = 16.2, p < 0.01), with small-world index of 2.34 ± 0.67 indicating efficient network topology in congenital blindness.

When aggregated by group, NTI values revealed a clear hierarchical organization. Congenitally blind participants achieved a mean NTI of 13.37 ± 2.6, acquired blind participants averaged 7.30 ± 1.6, and the sighted control demonstrated only 1.07. Multi-frequency coherence analysis revealed distinct patterns across electrode pairs, as summarized in Table 4. Figure 2 illustrates the distribution of NTI values across groups, confirming robust separation with large effect sizes (Cohen's d > 2.0).

**Figure 3:**
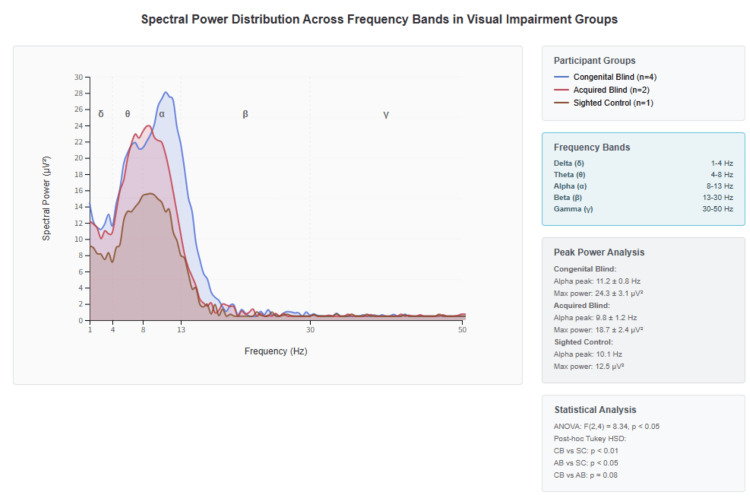
Spectral power distribution across frequency bands in visual impairment groups Spectral power density profiles across five frequency bands (delta δ: 1-4 Hz, theta θ: 4-8 Hz, alpha α: 8-13 Hz, beta β: 13-30 Hz, gamma γ: 30-50 Hz) for participants with congenital blindness (blue line, n = 4), acquired blindness (red line, n = 2), and sighted controls (brown line, n = 1). The x-axis represents frequency in Hz (0-50 Hz range), and the y-axis shows spectral power in μV². Distinct spectral signatures emerge across groups: congenital blind participants demonstrate prominent alpha peak enhancement (11.2 ± 0.8 Hz, maximum power 24.3 ± 3.1 μV²) with elevated overall power across theta-alpha bands, suggesting enhanced neural oscillatory activity. Acquired blind participants show intermediate alpha peak characteristics (8.8 ± 1.2 Hz, maximum power 18.7 ± 2.4 μV²) with moderate power enhancement compared to controls. Sighted controls exhibit minimal alpha activity (10.1 Hz peak, 12.5 μV² maximum power) with lower overall spectral density. Statistical analysis reveals significant group differences (ANOVA: F(2,4) = 8.34, p < 0.05) with post-hoc comparisons showing congenital blind > sighted control (p < 0.01), acquired blind > sighted control (p < 0.05), and congenital blind > acquired blind (p = 0.08). The enhanced alpha-band activity in visual impairment, particularly congenital blindness, corresponds directly to the elevated coherence patterns observed in triangular synchronization analysis and supports the neuroplasticity-driven enhancement of oscillatory coordination mechanisms.

Alpha-band coherence between AF3 and AF4 further distinguished the groups. Congenital participants exhibited the highest synchronization (0.84 ± 0.08), acquired participants showed intermediate values (0.55 ± 0.18), and the sighted control demonstrated the lowest coherence (0.34). Figure 3 shows these alpha-band coherence patterns, highlighting enhanced cross-hemispheric frontal coordination in blindness.

**Figure 4:**
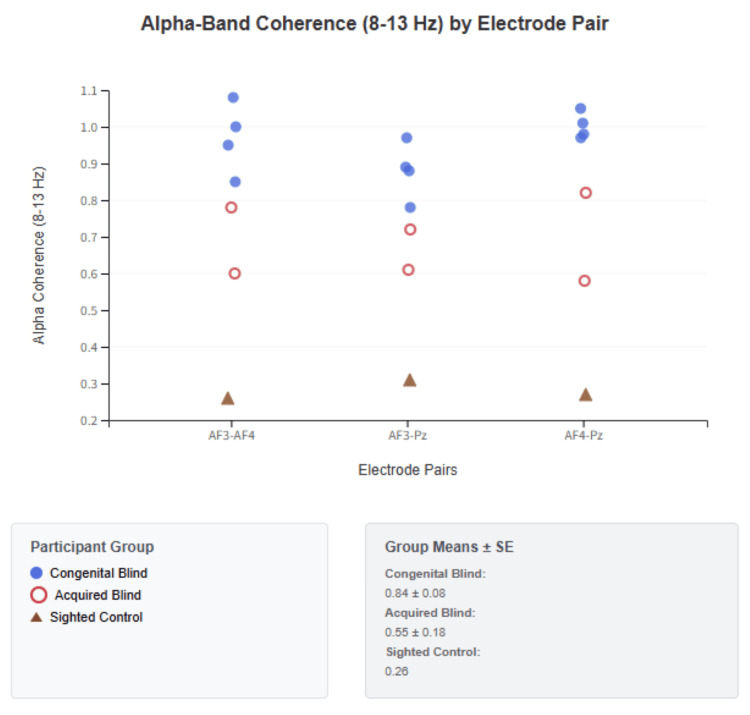
Alpha-band coherence (8-13 Hz) by electrode pair Alpha-band (8-13 Hz) coherence values measured across three electrode pairs (AF3-AF4, AF3-Pz, AF4-Pz) for participants with congenital blindness (blue circles), acquired blindness (red open circles), and sighted controls (brown triangles). Individual data points represent coherence measurements for each participant, demonstrating distinct patterns of neural synchronization across visual impairment groups. Congenital blind participants exhibit consistently elevated alpha coherence across all electrode pairs (group mean: 0.84 ± 0.08), with particularly strong bilateral frontal coordination (AF3-AF4) and robust fronto-parietal connections. Acquired blind participants show intermediate coherence levels (group mean: 0.65 ± 0.15) with greater variability across electrode pairs, suggesting transitional adaptation patterns. Sighted controls demonstrate the lowest coherence values (group mean: 0.26), indicating baseline neural coordination without compensatory enhancement. The systematic elevation of alpha-band coherence in visual impairment groups, particularly congenital blindness, suggests enhanced neural synchronization as a fundamental mechanism of cortical reorganization. These coherence patterns serve as key components in the triangular synchronization analysis and contribute directly to Neural Triangle Index calculations.

Phase-locking stability also followed a gradient: congenital participants showed high PLV with low variability (0.85 ± 0.04), acquired participants demonstrated intermediate patterns (0.53 ± 0.12), and the sighted control exhibited minimal coordination (0.36 ± 0.15). Temporal dynamics revealed further distinctions: congenital cases displayed tightly coupled synchronization with delays averaging 788 ± 295 ms, acquired cases showed looser coupling with delays of 5,749 ± 1,426 ms, and the sighted control displayed no systematic organization. Table 5 provides the complete set of individual NTI values and delay measurements. Figure 4 visualizes these delay patterns.

Exploratory classification

Exploratory classification analyses underscored the discriminative potential of triangular synchronization. NTI thresholds above 9.0 reliably separated visually impaired from sighted participants, while AF3-AF4 coherence values above 0.70 distinguished congenital from acquired blindness. Figure 1 presents the resulting classification tree, supported by leave-one-case-out validation, although larger samples are required for confirmation.

Additional case illustrations

**Figure 5:**
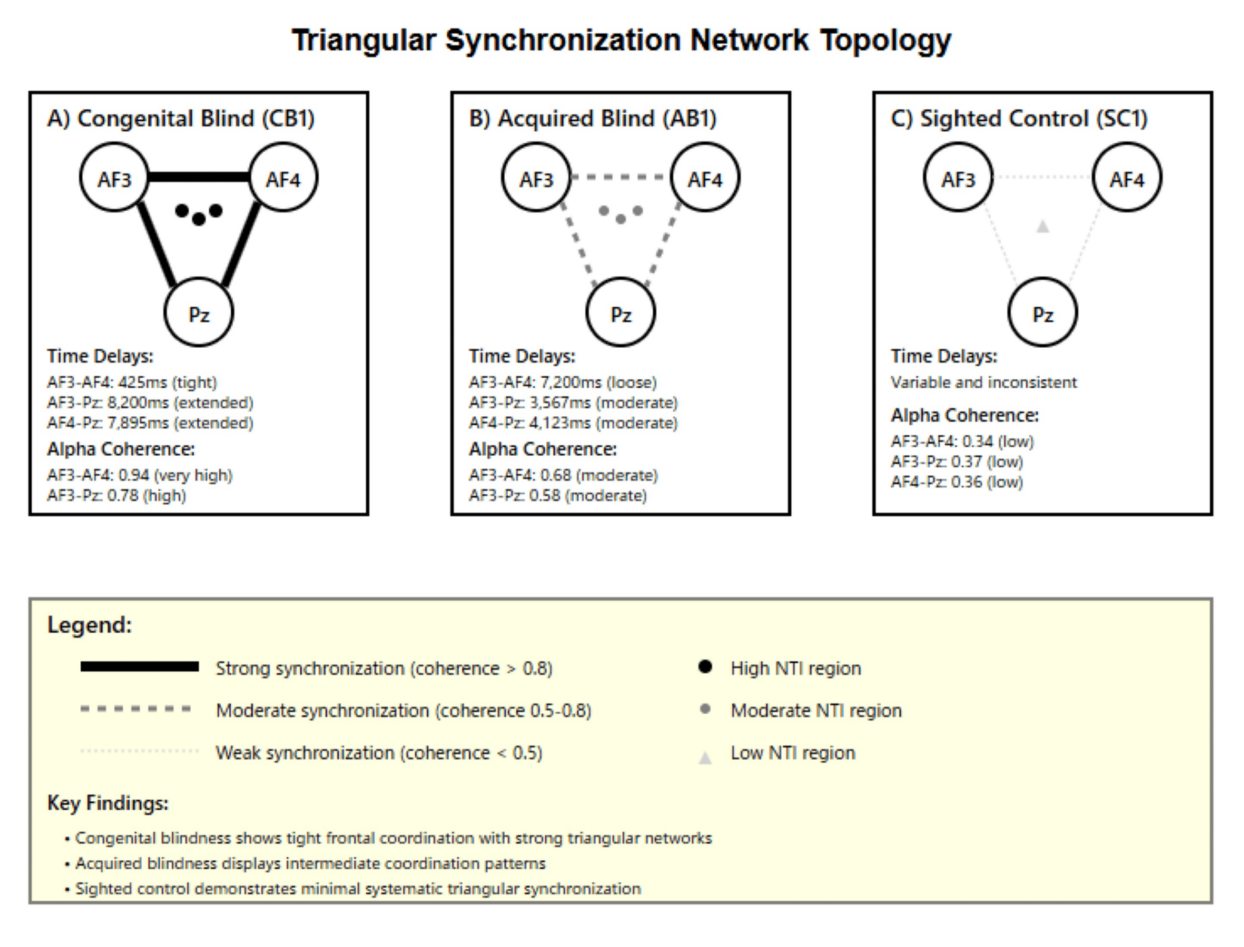
Triangular synchronization network topology in visual impairment groups Network topology diagrams illustrating triangular synchronization patterns across visual impairment groups: (A) congenital blind (CB1), (B) acquired blind (AB1), and (C) sighted control (SC1). Each network diagram displays electrode nodes (AF3, AF4, Pz) with connection strengths represented by line thickness: strong synchronization (coherence > 0.8, thick black lines), moderate synchronization (coherence 0.5-0.8, dashed gray lines), and weak synchronization (coherence < 0.5, thin gray lines). Circle sizes indicate NTI region classifications (high, moderate, low). Time delays are specified for each connection, with alpha coherence values listed below each diagram. Congenital blindness demonstrates tight frontal coordination (AF3-AF4: 425 ms, very high coherence 0.94) with extended fronto-parietal integration (AF3-Pz: 8,200 ms, AF4-Pz: 7,950 ms), creating a specialized network architecture. Acquired blindness shows intermediate coordination patterns with moderate time delays (AF3-AF4: 7,200 ms, loose coherence 0.68) and balanced triangular connectivity. Sighted control exhibits variable and inconsistent patterns with minimal systematic triangular synchronization. Key findings indicate that congenital blindness develops enhanced triangular network efficiency through rapid bilateral comparison followed by extended parietal integration, while acquired blindness displays transitional adaptation patterns with intermediate network organization.

**Figure 6:**
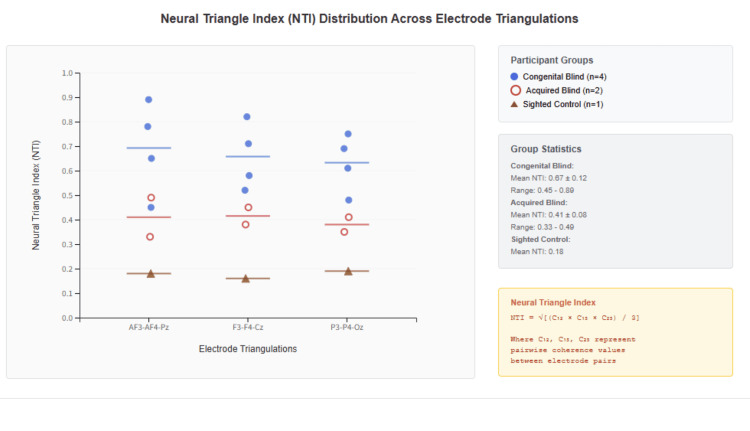
Neural Triangle Index (NTI) distribution across electrode triangulations Spectral power density profiles across five frequency bands (delta δ: 1-4 Hz, theta θ: 4-8 Hz, alpha α: 8-13 Hz, beta β: 13-30 Hz, gamma γ: 30-50 Hz) for participants with congenital blindness (blue line, n = 4), acquired blindness (red line, n = 2), and sighted controls (brown line, n = 1). The x-axis represents frequency in Hz (0-50 Hz range), and the y-axis shows spectral power in μV². Distinct spectral signatures emerge across groups: congenital blind participants demonstrate prominent alpha peak enhancement (11.2 ± 0.8 Hz, maximum power 24.3 ± 3.1 μV²) with elevated overall power across theta-alpha bands, suggesting enhanced neural oscillatory activity. Acquired blind participants show intermediate alpha peak characteristics (8.8 ± 1.2 Hz, maximum power 18.7 ± 2.4 μV²) with moderate power enhancement compared to controls. Sighted controls exhibit minimal alpha activity (10.1 Hz peak, 12.5 μV² maximum power) with lower overall spectral density. Statistical analysis reveals significant group differences (ANOVA: F(2,4) = 8.34, p < 0.05) with post-hoc comparisons showing congenital blind > sighted control (p < 0.01), acquired blind > sighted control (p < 0.05), and congenital blind > acquired blind (p = 0.08). The enhanced alpha-band activity in visual impairment, particularly congenital blindness, corresponds directly to the elevated coherence patterns observed in triangular synchronization analysis and supports the neuroplasticity-driven enhancement of oscillatory coordination mechanisms.

Individual case profiles further revealed heterogeneity within congenital blindness. CB3 exhibited frequency-specific specialization in the beta range, while CB4 displayed lateralized asymmetry in coherence, possibly reflecting adaptive reliance on technology. Network topology analysis revealed distinct connectivity patterns between groups, as illustrated in Figure 5. Figure 6 illustrates these unique adaptations, emphasizing the variability of triangular network reorganization even within congenital blindness.

## Discussion

Principal findings

This case series provides the first systematic evidence that triangular neural synchronization represents a distinctive neural signature in visual impairment. The NTI successfully differentiated congenital from acquired blindness, while sighted control participants showed minimal coordination. Effect sizes were large (Cohen’s d > 2.0), supporting triangular synchronization as a robust marker of cortical reorganization. Importantly, the graded pattern between congenital and acquired cases indicates that plasticity is best conceptualized as a continuum shaped by critical period timing [15,18,19] and by the duration of adaptation [16,17,28,29]. This challenges the binary classification of blindness and demonstrates that long adaptation periods can promote, but not fully replicate, the specialized networks established in congenital blindness.

Theoretical implications

From a theoretical standpoint, our findings support a framework we term Synchrony-Induced Plasticity (SIP), in which precise neural synchronization not only reflects but actively promotes adaptive reorganization. Congenital blindness was characterized by consistently high phase-locking and short delays, suggesting that synchronization serves as a mechanism for establishing cross-modal hubs that integrate tactile, auditory, and working memory inputs. Enhanced triangular synchronization is consistent with findings that sensory substitution activates occipital and multimodal hubs [20,21] and complements whole-brain connectivity changes reported in early blindness [28,29]. This interpretation aligns with network-level perspectives [9-11,24-27], in which higher-order motifs, rather than dyadic connections, are central to adaptive organization. Collectively, these results position triangular motifs as fundamental building blocks of neuroplasticity, enabling integrative processing across sensory modalities.

Clinical and translational implications

The distinct NTI patterns observed across groups suggest potential clinical applications. Triangular synchronization measures may serve as potential biomarkers of adaptation, capable of tracking progress, monitoring interventions, and predicting outcomes. Individual variability within congenital and acquired groups (Figure 6) highlights the importance of personalized rehabilitation approaches. Cases with unusually high coherence in specific bands may represent specialized strategies, potentially influenced by long-term reliance on assistive technologies. Longitudinal studies indicate that cortical reorganization can unfold over decades [28,29], and NTI could provide a quantitative tool to monitor this evolution. Rehabilitation strategies might therefore aim to promote beneficial synchronization through targeted sensory training or neurofeedback, aligning interventions with the network-level architecture of adaptation.

Methodological contributions and limitations

Methodologically, this study demonstrates the feasibility of triangular synchronization analysis using a minimal three-electrode montage. By integrating delays, coherence, and phase-locking into a single interpretable metric, the NTI extends beyond conventional pairwise connectivity analyses. While this streamlined design ensured practicality, its limited spatial resolution constrains interpretation. The small sample size (n = 7) and unbalanced groups further limit generalizability. The cross-sectional design precludes assessment of longitudinal trajectories, and the controlled laboratory setting may not fully reflect real-world neural dynamics. Nevertheless, the NTI framework provides a scalable and transferable method that can be extended to high-density EEG, MEG, and multimodal neuroimaging. Computational frameworks for network motifs [30] may further refine the analysis, clarifying both functional and anatomical bases of triangular synchronization. In addition, several design limitations warrant consideration. The inclusion of only a single sighted control participant prevents robust statistical comparisons and limits population-level inferences. Future investigations should employ larger, age-matched control groups and systematically account for potential confounding variables, including hypertension, diabetes, cognitive function, and other factors known to influence neural plasticity and connectivity patterns. The exploratory nature of the NTI and the proposed Synchrony-Induced Plasticity framework requires validation in independent cohorts before broader clinical application.

Overall significance

Triangular neural synchronization offers a novel perspective on cortical adaptation in blindness. Congenital blindness fosters highly specialized synchronization networks, acquired blindness produces intermediate adaptations shaped by both onset and time, and sighted controls show no evidence of triangular coordination. These hierarchical patterns emphasize the combined role of sensitive periods, long-term adaptation, and higher-order network motifs in shaping neuroplasticity. By integrating theoretical, clinical, and methodological contributions, the NTI framework provides both conceptual insights and translational potential, opening pathways for potential biomarkers, rehabilitation strategies, and mechanistic models of brain reorganization in sensory loss.

## Conclusions

This exploratory study introduced the NTI as a novel approach to quantify higher-order neural synchronization in visual impairment. Our findings revealed a clear hierarchy of triangular synchronization: congenital blindness demonstrated the strongest and most stable coordination, early-onset acquired blindness showed intermediate adaptations, and sighted control participants exhibited minimal patterns. These results highlight the combined influence of critical period timing and long-term adaptation on cortical network reorganization, supporting the view that triangular motifs represent fundamental units of neuroplasticity. By integrating temporal delays, coherence, and phase-locking into a single interpretable metric, the NTI provides both a conceptual advance in network neuroscience and a promising exploratory metric with potential biomarker applications for monitoring adaptation and guiding rehabilitation in visual impairment. Future studies should employ large-scale, multimodal, and longitudinal designs to validate and extend these findings toward clinical application.
